# The network structural entropy for single-cell RNA sequencing data during skin aging

**DOI:** 10.1093/bib/bbae698

**Published:** 2025-01-05

**Authors:** Zhilong Liu, Hai Lin, Xiang Li, Hao Xue, Yuer Lu, Fei Xu, Jianwei Shuai

**Affiliations:** Department of Physics, Xiamen University, No. 422, Siming South Road, Xiamen, Fujian, 361005, China; Oujiang Laboratory (Zhejiang Lab for Regenerative Medicine, Vision and Brain Health), No. 999, Jinshi Road, Yongzhong Street, Longwan District, Wenzhou, Zhejiang, 325000, China; Wenzhou Institute, University of Chinese Academy of Sciences, No. 1, Jinlian Road, Longwan District, Wenzhou, Zhejiang, 325000, China; Department of Physics, Xiamen University, No. 422, Siming South Road, Xiamen, Fujian, 361005, China; Department of Computational Biology, Cornell University, 110 Biotechnology Building, Ithaca, 14853 NY, United States; Oujiang Laboratory (Zhejiang Lab for Regenerative Medicine, Vision and Brain Health), No. 999, Jinshi Road, Yongzhong Street, Longwan District, Wenzhou, Zhejiang, 325000, China; Wenzhou Institute, University of Chinese Academy of Sciences, No. 1, Jinlian Road, Longwan District, Wenzhou, Zhejiang, 325000, China; Department of Physics, Anhui Normal University, No. 189 Jiuhua South Road, Wuhu, Anhui, 241002, China; Oujiang Laboratory (Zhejiang Lab for Regenerative Medicine, Vision and Brain Health), No. 999, Jinshi Road, Yongzhong Street, Longwan District, Wenzhou, Zhejiang, 325000, China; Wenzhou Institute, University of Chinese Academy of Sciences, No. 1, Jinlian Road, Longwan District, Wenzhou, Zhejiang, 325000, China

**Keywords:** aging, single-cell RNA sequencing, gene regulatory networks, network structural entropy, cellular heterogeneity

## Abstract

Aging is a complex and heterogeneous biological process at cellular, tissue, and individual levels. Despite extensive effort in scientific research, a comprehensive understanding of aging mechanisms remains lacking. This study analyzed aging-related gene networks, using single-cell RNA sequencing data from >15 000 cells. We constructed a gene correlation network, integrating gene expressions into the weights of network edges, and ranked gene importance using a random walk model to generate a gene importance matrix. This unsupervised method improved the clustering performance of cell types. To further quantify the complexity of gene networks during aging, we introduced network structural entropy. The findings of our study reveal that the overall network structural entropy increases in the aged cells compared to the young cells. However, network entropy changes varied greatly within different cell subtypes. Specifically, the network structural entropy among various cell types may increase, remain unchanged, or decrease. This wide range of changes may be closely related to their individual functions, highlighting the cellular heterogeneity and potential key network reconfigurations. Analyzing gene network entropy provides insights into the molecular mechanisms behind aging. This study offers new scientific evidence and theoretical support for understanding the changes in cell functions during aging.

## Introduction

The emergence of complex biological processes is typically not caused by a single molecule, but results from the disruption of cellular signaling or the multifaceted responses to the external environment, driven by dynamic changes in the interactions between numerous molecules [1]. In biological networks, the interactions between molecules constitute the edges of the network and form the basis of biological functions and signal transduction [2]. Therefore, it is crucial to explore the dynamic changes in molecular interactions and gain a comprehensive understanding of molecular networks. The transcriptional regulation of genes underpins all fundamental cellular processes. Interactions between genes form gene regulatory networks (GRNs) that control cell identity and fate determination, and play significant roles in the development of various diseases [3]. Consequently, the changes in GRN may alter the state or the function of cells [4–6], thereby triggering the onset of diseases [7, 8]. Constructing GRNs helps researchers to better understand the interactions between genes.

To construct GRNs, researchers have exerted considerable effort and applied a variety of modeling methods [9–11]. Traditionally, scientists often focused on analyzing a limited number of signaling pathways [12]. However, with the rise of systems biology, the scope of the research has expanded to the entire genome. In particular, the advancement of single-cell transcriptomics technology has provided us with an unprecedented ability to observe gene expression changes across different cell types and organs [13–15]. Research on biological systems generally focuses on exploring significant variations in gene expression during biological processes [16]. However, studies have shown that even small changes in the expression of non-differentially expressed genes can lead to substantial biochemical effects and play a key role in a variety of biological functions [17–19]. Therefore, network-based exploration of differential molecular interactions rather than differential expression can reveal the underlying dynamic changes in molecular regulatory relationships, thereby better characterizing the transformation of biological functions [20]. Currently, researchers have developed a variety of models, including Bayesian network models [21, 22], graphical Gaussian models [23], and correlation networks [24], to directly extract information of gene interactions from expression profiles and determine potential regulatory relationships [25]. These models provide a comprehensive perspective for understanding the mechanisms of complex disease pathogenesis from a network-based viewpoint [26–29].

Aging is a complex and multifactorial biological process associated with various diseases such as malignancies, diabetes, cardiovascular diseases, and neurodegenerative disorders [30]. The occurrence of aging is profoundly influenced by intracellular gene regulation [31–33]. With the application of single-cell transcriptomics [34–37], researchers have extensively explored gene expression changes during aging [38, 39]. Although existing studies have partially revealed ongoing changes in gene expression and signaling pathways during aging [40], our understanding of this complex process remains limited. Traditional methods often focus on a single aspect of gene expression or gene networks, which restricts understanding of the mechanisms of aging. Therefore, an integrated approach that considers both gene expression and network information is needed to gain a more comprehensive and in-depth understanding of the aging.

In this study, we first adopted a single-cell gene importance ranking method to construct an aging-related single-cell weighted gene network [41, 42]. By constructing a gene correlation network based on gene expression correlations at the single-cell level and integrating gene expression information into the weights of the network edges, we further utilized a random walk model to rank the importance of genes, generating a gene importance matrix (GIM). Additionally, we introduce the concept of network structural entropy to quantify the complexity of aging single-cell gene networks, thereby providing a new perspective for understanding the structural and dynamic changes of gene networks during aging. This study aims to construct a gene network through aging-related single-cell data and explore the potential value of single-cell gene importance ranking and network structural entropy in aging research, with the expectation of providing new scientific evidence and theoretical support for a deeper understanding of aging mechanisms.

## Materials and methods

### Data

During the aging process, the skin, which is the largest organ of the human body, undergoes various changes such as thinning, reduced elasticity, wrinkles, and pigmentation. As a highly complex and heterogeneous organ composed of various cells and tissues [43], the aging of the skin is also complex and heterogeneous [44]. To investigate the intrinsic mechanisms of skin aging, in this study, we utilized the single-cell RNA sequencing (scRNA-seq) dataset by Llorenç *et al*. [45], which specifically excludes the interference of external environmental factors such as photoaging. The dataset comprises 15 457 cells of 13 different types (Table 1), collected from five independent skin samples from the inguinal iliac region of male donors of different ages.

**Table 1:** Details of the dataset

Cell type	Number of cells	Number of young cells	Number of old cells
Diff. keratinocytes	1399	130	1269
EpSC and undiff. progenitors	1187	182	1005
Erythrocytes	310	272	38
Lymphatic EC	294	65	229
Macrophages + DC	2228	680	1548
Melanocytes	123	34	89
Mesenchymal	594	347	247
Pericytes	1220	808	412
Pro-inflammatory	1793	592	1201
Secretory-papillary	1675	454	1221
Secretory-reticular	1886	399	1487
T cells	1281	1054	227
Vascular EC	1467	437	1030
Total	15 457	5454	10 003

### Data preprocessing

In single-cell data analysis, preprocessing the data is a crucial step to ensure data accuracy and reliability. In this study, the raw data have passed quality control. We further normalized and logarithmically transformed the dataset and obtained the preprocessed Gene Expression Matrix (GEM).

### Construction of single-cell gene correlation networks

Previous research has demonstrated that studying cell-specific networks using single-cell data is effective for analyzing cellular heterogeneity and complexity [46]. Therefore, we use single-cell data to construct gene correlation networks.

**Figure 1:**
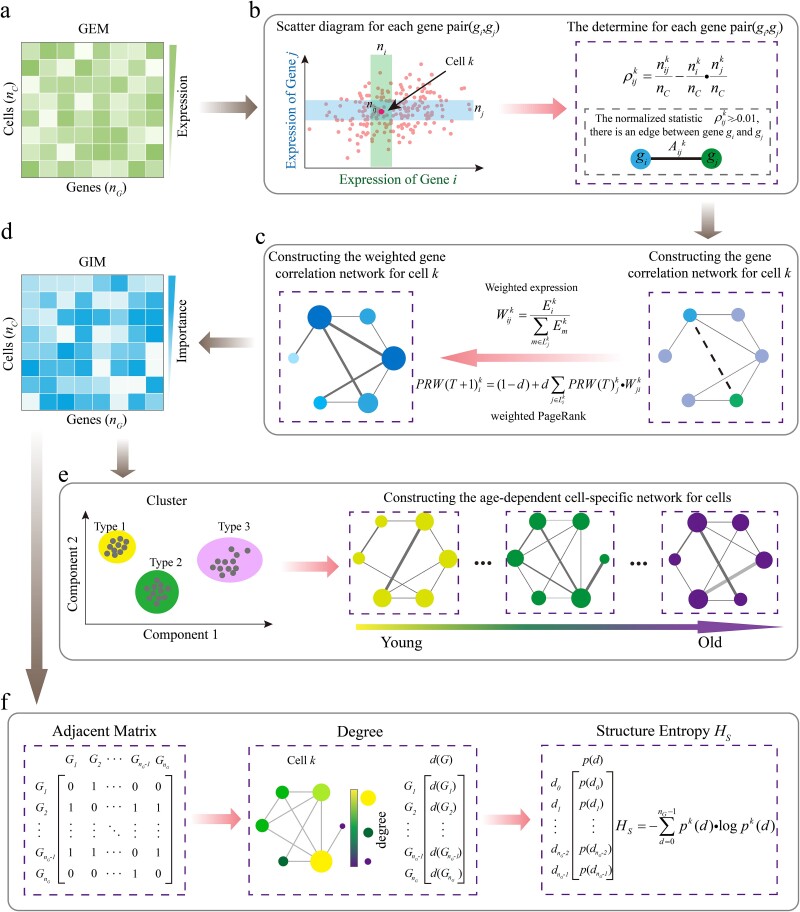
Schematic diagram of constructing the single-cell gene correlation network. (a) Data preprocessing. The preprocessed single-cell GEM consists of ${n}_C$ cells and *n_C_* genes. (b) Gene correlation identification. Scatter plots are created for every gene pair in the matrix, resulting in ${n}_G\ast \left({n}_G-1\right)/2$ scatter plots, each containing ${n}_C$ points (cells). In the scatter plot for genes *i* and *j*, the neighborhoods around cell *k* are represented by two boxes for genes *i* and *j*, respectively. The number of cells within the boxes are ${n}_i^k$ and ${n}_j^k$, with ${n}_{ij}^k$ being the number of cells in the intersection. Based on this, the independence statistic ${\rho}_{ij}^k$ and correlation ${A}_{ij}^k$ are calculated. (c) Construction of weighted gene correlation network. For each cell *k*, a network is constructed based on the gene correlations matrix *A*. The weight of the edge between genes *i* and *j* is calculated based on gene expression levels. A total of ${n}_C$ networks are constructed. (d) Generation of single-cell GIM. A random walk model is constructed for each gene-weighted correlation network, and a ranking algorithm is used to determine the importance ranking of genes in the cells, ultimately resulting in a GIM that includes all cells. (e) Construction of cell-type-specific networks with age variation. (f) Calculation of network structural entropy. The adjacency matrix of marker genes and the degree *d*(*G*) of corresponding gene nodes are obtained, along with the probability *p*(*d*) of each degree, to compute the network structural entropy *H_S_*.

The schematic representation of the preprocessed GEM is shown in Fig. 1a. The element of GEM, ${E}_i^k$, denotes the expression level of gene *i* in cell *k* in the GEM. To construct a gene correlation network, we first need to identify the correlations between gene pairs. Typically, researchers use statistical methods to quantify these correlations [42, 47]. In this process, based on the independence assumption between gene pairs, we calculated GEM for all gene pairs. Taking gene *i* and gene *j* as an example, we mapped the expression value of gene *i*, ${E}_i$, and the expression value of gene *j*, ${E}_j$, of each cell into a two-dimensional gene expression space, forming a scatter plot, as shown in Fig. 1b (left side). In this scatter plot, each point represents an individual cell, with its position determined by the expression levels ${E}_i$ and ${E}_j$ of genes *i* and *j*, respectively.

For further analysis, we define two neighborhoods around cell *k*, corresponding to the expression values neighborhoods of gene *i* and gene *j*, respectively. Here, ${n}_i^k$ represents the number of cells where the expression level of gene *i* is close to the expression level ${E}_i^k$ of gene *i* in cell *k*; ${n}_j^k$ represents the number of cells where the expression level of gene *j* is close to the expression level ${E}_j^k$ of gene *j* in cell *k*; ${n}_{ij}^k$ represents the number of cells in the intersection of these two neighborhoods, i.e. those cells whose expression levels of both genes *i* and *j* are close to the corresponding gene expression levels in cell *k*. In cell *k*, the independence index statistic ${\rho}_{ij}^k$ for genes *i* and *j* is given by the following formula:


(1) \begin{equation*} {\rho}_{ij}^k=\frac{n_{ij}^k}{n_C}-\frac{n_i^k}{n_C}\cdot \frac{n_j^k}{n_C}, \end{equation*}

where ${n}_C$ represents the total number of cells in the GEM. For simplification, ${n}_i^k$ and ${n}_j^k$ are preset to be 0.1${n}_C$. Previous studies [47] have shown that the statistic ${\rho}_{ij}^k$ can be used to analyze gene associations *A_ij_^k^* at the single-cell level, where *A_ij_^k^* is defined as shown in Equation (S1). In the process of constructing the gene correlation network, the existence of each edge indicates that the statistical independence index ${\rho}_{ij}^k$ between gene *i* and gene *j* is greater than or equal to the significance level, 0.01, as shown in Fig. 1b (right). In this study, the significance level, set consistently at 0.01 as the threshold, serves as the criterion for assessing the correlation between two genes within individual cells, aligning with previous relevant reports [18, 47, 48]. By repeating this process for all gene pairs and all cells, we can ultimately construct ${n}_C$ gene correlation networks for ${n}_C$ cells based on the gene correlations matrix *A*.

### Gene expression–weighted gene correlation edges

Although gene networks constructed through correlations can reveal interaction patterns between genes, this method may have limitations. It might overlook the quantitative information of gene expression levels, thereby leading to the loss of critical expression details, as shown in Fig. 1c (right side). To remedy this deficiency, we introduce the concept of weighted edges, assigning gene expression levels as weights to the connections between gene pairs, thereby obtaining a weighted single-cell gene correlation network, as shown in Fig. 1c (left side). When there is a correlation between genes *i* and *j* in cell *k*, the corresponding edge weight ${W}_{ij}^k$ can be defined as


(2) \begin{equation*} {W}_{ij}^k=\frac{E_i^k}{\sum_{m\in{L}_j^k}{E}_m^k}, \end{equation*}

where ${E}_m^k$ represents the expression level of gene *m* and ${L}_j^k$ denotes the set of neighboring genes of gene *j* in cell *k*. For any gene *i* connected to gene *j* within the gene set, we not only identify whether there is a correlation but also assign a quantitative weight to the correlation edge based on the expression level of gene *i*. Additionally, it is important to note that an unsupervised method is used to analyze the preprocessed data, which means that we reveal the intrinsic structure of the data itself, rather than being based on any specific model or expected outcome.

### Quantifying gene node importance using random walks

Next, in the analysis of single-cell gene correlation networks, we employ the PageRank algorithm to evaluate the importance of genes [49]. This algorithm relies on two fundamental assumptions: (i) the quantity assumption, which posits that the importance of a web page increases with the number of inbound links it receives from other pages; and (ii) the quality assumption, which posits that the importance of a web page also increases when it is linked by multiple high-quality pages. Specifically, the PageRank algorithm quantifies the importance of each gene node by calculating its score through the formula (S2). In a gene network, if a gene node has more associated edges, or is connected to more than one key gene node, it usually has a higher PageRank score, indicating that it is more biologically important. However, the traditional PageRank method does not fully consider the expression values of the gene nodes. To further incorporate the information of the expression level of a gene, we introduce a weighting mechanism to balance and enhance the assessment of node importance. The weighted PageRank value ${PRW}_i^k$ considering the gene expression weight ${W}_{ji}^k$ is calculated using the following formula:


(3) \begin{equation*} PRW{\left(T+1\right)}_i^k=\left(1-d\right)+d\sum_{j\in{L}_i^k} PRW{(T)}_j^k\cdot{W}_{ji}^k, \end{equation*}

where ${W}_{ji}^k$ is obtained from Equation (2) and represents the weight of gene *j* associated with gene *i* in cell *k*. $PRW{\left(T+1\right)}_i^k$ denotes the PageRank value of gene *i* in cell *k* at iteration *T* + 1, which integrates the gene expression weight ${W}_{ji}^k$. $PRW{(T)}_i^k$ represents the PageRank score of gene *j* in cell *k* at iteration *T*. The iteration stops when the number of iterations reaches 100 or the convergence accuracy reaches 1.0e−6. By employing this method, we can calculate the GIM, as shown in Fig. 1d. Given that we have constructed a unique gene network for each cell, to ensure comparability of gene networks across different cell types, we integrate and normalize the gene networks of all cells of a specific cell type to form a representative network by Equation (S3), as shown in Fig. 1e*.*

### Structural entropy for characterizing complexity of gene correlation networks

To gain a deeper understanding of the complex characteristics of gene networks during aging, we employed the method of quantifying network structural entropy [50–52], as illustrated in Fig. 1f. First, in cell *k*, based on the gene correlation network constructed from the gene correlation matrix A, we calculated the degree *d*(*G*) of each gene node. Then, we counted the number of nodes ${n}^k$(*d*) for each degree in the network. Subsequently, we divided the number of nodes for each degree by the total number of gene nodes ${n}_G$ in the network to obtain the degree probability *p^k^*(*d*). The degree probability is defined as


(4) \begin{equation*} {p}^k(d)=\frac{n^k(d)}{n_G}. \end{equation*}

Then, based on the distribution characteristics of these degrees, we quantified the network structural entropy. The gene network structural entropy ${H}_S^k$ for cell *k* is calculated using the following formula:


(5) \begin{equation*} {H}_S^k=-\sum_{d=0}^{n_G-1}{p}^k(d)\cdot \log{p}^k(d). \end{equation*}

## Results

### The gene importance matrix more accurately reveals single-cell age differences

**Figure 2:**
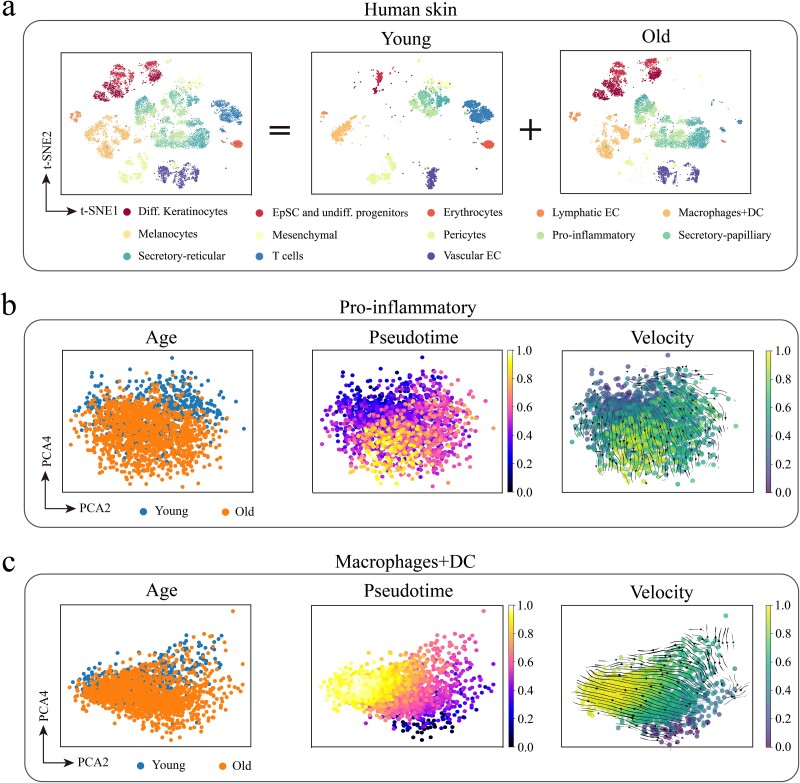
Visualization of human skin scRNA-seq data. (a) t-SNE visualization of 13 cell types in human skin. (b) Age-dependent changes in gene expression of pro-inflammatory cells. (c) Age-dependent changes in gene expression of macrophages + DC.

Based on the preprocessed GEM, we performed a detailed analysis of the cellular composition of human skin and identified 13 different cell types, as shown in Fig. 2a (left side). Then, we conducted a comparative analysis of the cell types captured in young and aged cells, with results presented in Fig. 2a (middle) and Fig. 2a (right). The results indicate that all 13 cell types were captured in both young and aged cells.

Aging is a complex physiological process, one of the characteristics of which is a persistent inflammatory response [53–55]. The senescence-associated secretory phenotype is a hallmark of cellular senescence, which functions by promoting local inflammation and recruiting immune cells. In this process, pro-inflammatory cells and macrophages + DC cells play unique roles [56]. Pro-inflammatory cells generally refer to those cells that play a critical role in inflammatory responses by secreting pro-inflammatory cytokines and chemokines, which initiate and propagate inflammation. In contrast, the role of macrophages + DC cells in inflammatory diseases is more complex. Macrophages, depending on their function and activation status, can execute either pro-inflammatory or anti-inflammatory actions through cytokine secretion, and play a key role in immune responses and immune regulation through phagocytosis and antigen presentation to defend against pathogen invasion [57, 58]. However, aging macrophages and dendritic cells may experience physiological gene regulation changes and functional impairments [59–62]. Therefore, understanding the changes in these cells during aging is crucial for the prevention and treatment of related diseases. Based on this, we analyzed pro-inflammatory cells and macrophages + DC cells, and the results are shown in Fig. 2b and2c. Figure 2b illustrates the gene expression differences in pro-inflammatory cells between young and aged skin. On the left side of the figure, the cluster analysis distinguishes the cells in the young and old groups, forming two distinct clusters. In the middle of the figure, CytoTRACE was used to perform pseudotime trajectory analysis [63], which simulates the continuous change of cell state and thus infers the developmental trajectory of the cell with age. The results show that the age distribution of pro-inflammatory cells is consistent with their developmental trajectory, i.e. young cells eventually develop into senescent cells. Additionally, single-cell RNA velocity analysis was also performed to infer cell developmental trajectories [64]. The results of RNA velocity analysis on the right side of Fig. 2b further verify the direction and rate of cell state change, revealing the dynamic process of young cells transitioning into aging cells. This provides strong support for understanding the dynamic changes of cells during aging. Similarly, we performed the same analysis on macrophages + DC cells and obtained consistent conclusions, and the results are depicted in Fig. 2c.

**Figure 3:**
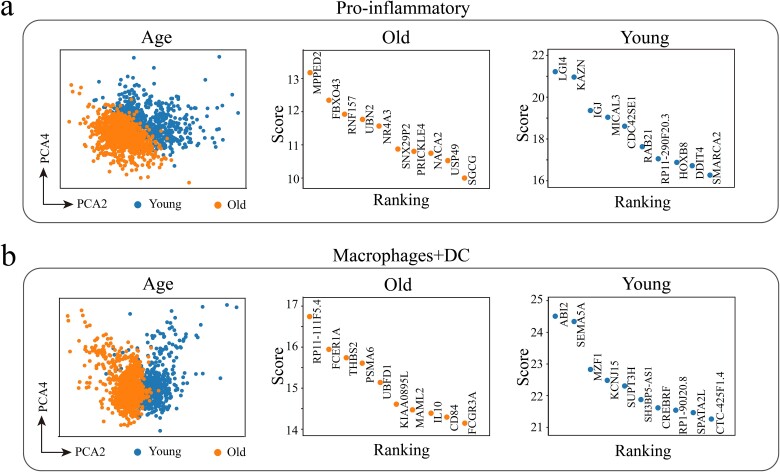
Visualization analysis of single-cell GIM. (a) Pro-inflammatory cells. (b) Macrophages + DC cells. Left: distribution of young and aging cells in the PC2 versus PC4 combination space in the PCA. Middle and right: top 10 marker genes with the most significant expression differences between young and aging cells identified by *t*-test, along with their ranking.

Next, we evaluated the performance of the GIM, with the results presented in Fig. 3. Compared to the analysis results in Fig. 2, which used only gene expression information, the new method integrates gene expression and gene correlation network data, demonstrating superior performance. Specifically, the visualization results in Fig. 3a (left) and Fig. 3b (left) clearly distinguish between young and aging cells in pro-inflammatory cells and macrophages + DC cells, with more tightly clustered cell groups. The visualization results of other cell types are shown in Figs. S1 and S2 in the supplementary materials. Furthermore, to quantitatively compare the performance of GIM and GEM in distinguishing young and aging cells, we used Adjusted Rand Index (ARI) and Normalized Mutual Information (NMI) as evaluation metrics and performed the analysis for each cell type [48, 65]. As shown in Fig. S3, the results indicate that GIM consistently outperforms GEM across all combinations of principal components (PCs) considered. Notably, GIM performs particularly well in the combined space of PC2 and PC4, which is superior to other combinations of principal components. In addition, we also compared the Gene Correlation Network Degree Matrix method [47] and evaluated it using ARI, Fowlkes–Mallows Index, and NMI. Our results further confirm the superior performance of GIM, as shown in Fig. S4.

The results of Fig. 3a (right) and Fig. 3b (right) particularly emphasize the key roles of specific genes in the aging. We found that the gene MPPED2 and gene FBXO43 play important roles in the regulation of aging pro-inflammatory cells. Studies have confirmed that the gene MPPED2 plays a crucial role in cell proliferation, migration, and invasion [66]. At the same time, the gene FBXO43 is also involved in the regulation of the cell cycle [67], and its function may be related to the increased proliferation of pro-inflammatory cells such as Th17 during aging [68]. Similarly, we found that the gene FCER1A plays a crucial role in aging macrophages + DC cells. The protein encoded by the gene the function of FCER1A in dendritic cells is critical for the regulation of immune responses [69]. Dysfunction of FCER1A during aging may lead to abnormal immune responses, affecting pathogen clearance and inflammation control. For young macrophages + DC cells, the gene SEMA5A shows its unique role, with its encoded protein being highly related to cell growth and development [70]. Additionally, SEMA5A can regulate the proliferation of immune cells, thereby modulating immune responses [71]. Furthermore, if a gene exhibits significant differences between sample and control sample at the network level rather than the gene expression level, we refer to this gene as a “dark” gene [47]. Based on the GIM, we revealed several “dark” genes in pro-inflammatory cells and macrophages + DC cells, as shown in Fig. S5. Although at the level of gene expression, these genes did not show differences between different cell groups and showed low expression levels, they may play an important role in cell biological functions. For example, MT1F has been confirmed to be associated with cell migration and invasion [72].

### Gene network analysis reveals cell specificity

**Figure 4:**
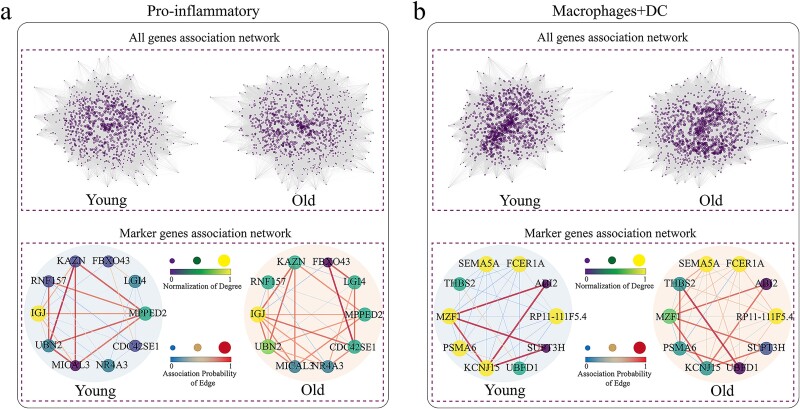
Integrated gene network of young and aging cells. (a) Pro-inflammatory cells. (b) Macrophages + DC cells. The upper panel displays the complete gene network containing all genes, where nodes represent genes and the size of the nodes reflect the relative size of the node degree. Network edges represent the correlation between gene pairs, and the weight of the edge is the ratio of the sum of the number of cells with correlated gene pairs in all cell networks to the number of all cells. That is, the thickness and color of the network edges represent the strength of the association and probability between genes. The lower side shows the marker gene network.

The GIM not only reveals differences in gene expression among different cell types but also uncovers disparities in gene networks between different cell types. To further explore cellular heterogeneity, we constructed a mixed network that integrates young and aging marker genes. This strategy allows us to simultaneously investigate the heterogeneity between different cell types, as well as the heterogeneity within the same cell type, by comparing the young cell network and the aging cell network, and examining their dynamic changes over time. Figure 4 illustrates the weighted gene correlation networks of pro-inflammatory cells and macrophages + DC cells. Due to cellular heterogeneity, these networks were normalized to ensure comparability among gene networks from different cells. However, as shown in Fig. 4a (upper), despite normalizing the full gene networks of young and aged pro-inflammatory cells, the complexity of the network still makes it challenging to intuitively display information about nodes and edges. Therefore, to overcome this challenge, we simplified the network structure by focusing on the top 10 marker genes in young and aged cells. Figure 4a (bottom) displays the network of the pro-inflammatory cells. By comparing the marker gene networks of young and aged pro-inflammatory cells, significant differences are observed.

In aging pro-inflammatory cells, the degree of some nodes remained relatively unchanged, possibly indicating that gene interactions remain stable during aging. Additionally, the interaction probabilities between nodes also changed, which may indicate a restructuring of the gene network in aging cells. For example, the increased interaction probability between CDC42SE1 and FBXO43 may be related to the proliferation of pro-inflammatory cells in aging cells [67]. At the same time, the decreased interaction probability between the RNF157 and MPPED2 genes may be related to the regulation of inflammatory and immune responses [73].

For macrophages + DC cells, Fig. 4b presents similar results. Compared to young cells, aging macrophages + DC cells exhibit significantly different network characteristics, where the degree of some marker gene nodes decreases, which may reflect a reduction or loss of interactions between these genes during aging, such as reduced phagocytic and antigen-presenting capabilities. Nevertheless, we also observed that the interaction probabilities between certain genes increased in aging macrophages + DC cells. Specifically, the interaction probability between gene THBS2 and gene UBFD1 significantly increased in aging cells. THBS2, an extracellular matrix glycoprotein, may influence immune cell migration and localization by regulating interactions between immune cells and the extracellular matrix [74]. The gene UBFD1 participates in the degradation of intracellular proteins [75], and its expression in immune cells is closely linked to cell cycle regulation and stress responses. The enhanced interaction between these specific genes may be related to extracellular matrix remodeling, adjustment of immune cell migration, and maintenance of intracellular protein balance in macrophages + DC cells.

### Quantifying cellular network complexity through gene network entropy

Network structural entropy, as a key indicator of the complexity of networks, provides a new perspective for understanding changes in gene interaction patterns during aging. In network analysis, entropy holds significance at both macroscopic and microscopic levels: at the micro level, it focuses on the connectivity characteristics of individual nodes; at the macro level, it emphasizes the heterogeneity of the entire network [50]. This study quantified the entropy values by analyzing the degree distribution of gene nodes. Firstly, we constructed weighted gene networks for each cell of every cell type. Based on these networks, we then generated gene link matrices for each cell. Subsequently, we calculated the degree of each gene node in each cell and analyzed the frequency distribution of the degrees in each cell. We then normalized these frequencies to form the probability distribution of the degrees. Finally, based on this probability distribution, we calculated the network structural entropy of the gene network for each cell to quantify the complexity of the network.

**Figure 5:**
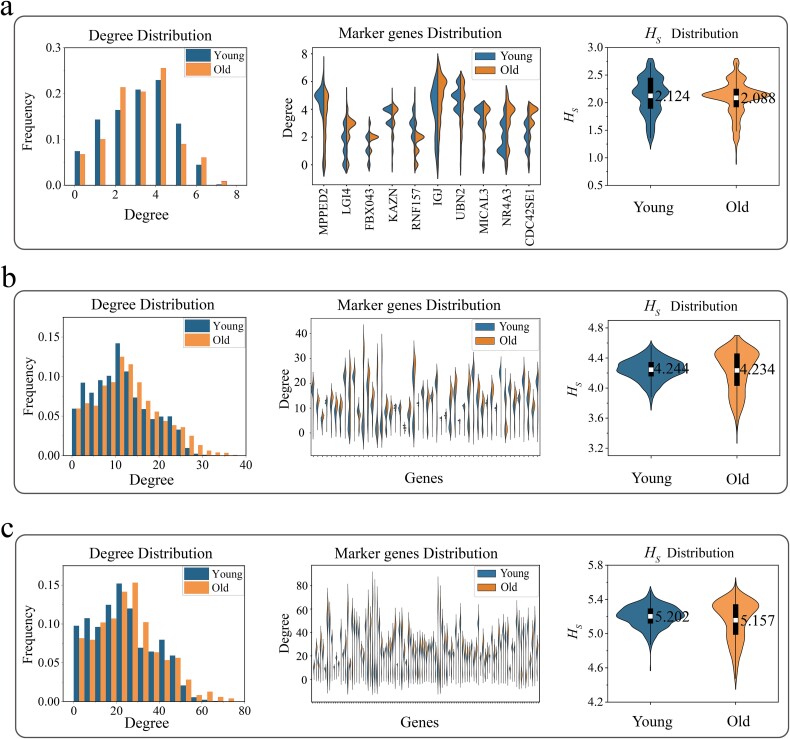
Gene network structural entropy of pro-inflammatory cells. (a) Top 10 marker genes. (b) Top 50 marker genes. (c) Top 100 marker genes. The left panel shows the frequency of the degree of gene nodes in the network, the middle panel displays the distribution of the degree of gene nodes, and the right panel shows the distribution of gene network structural entropy between young and aging cells. The white box and number in the violin plots represent the mean value.

By applying this method, we delved into the structural characteristics of gene networks in pro-inflammatory cells and presented the distribution of node degrees and entropy in networks with varying numbers of marker genes in Figs. 5a, 5b, and5c. The histograms on the left show the overall degree distribution of the gene networks in young and aging cells. The results indicate that the degree distribution in aging cells is similar to that in young cells. The shape of the degree distribution, as well as the mean and standard deviation, remain relatively stable during the aging, as shown in Fig. S6a. This suggests that the GRN in pro-inflammatory cells remains highly stable and consistent during aging. The middle part of the figure details the degree distribution of specific gene nodes. For example, the degree distribution of the gene NR4A3 and gene IGJ node in aging cells is similar to that in young cells. This observation indicates that the gene NR4A3 and gene IGJ plays a crucial role in regulating inflammatory responses in both aging and young cells [76]. This finding aligns with the phenomenon of inflammatory responses in aging cells. It suggests that pro-inflammatory cells have an inherent stability, maintaining a certain gene expression pattern during aging.

The right side of Fig. 5 shows the distribution of network entropy in the gene networks of young and aging cells. The results indicate that there is no significant difference in the average structural entropy of gene networks between young and aging pro-inflammatory cells, and the average entropy of aging cells remains stable. The white squares in the center of the violin plots clearly indicate the positions of the averages. This indicates that the gene network structure of pro-inflammatory cells is basically stable and maintains its pro-inflammatory function. Studies have shown that senescent cells maintain a higher inflammatory response. Additionally, studies have shown that a moderate inflammatory response is important for clearing pathogens and damaged tissues and promoting tissue repair [77]. Aging cells can help to maintain tissue homeostasis by secreting cytokines and growth factors [78]. This adaptability is crucial not only for the survival of the cells themselves but also for the health and function of the entire organism.

**Figure 6:**
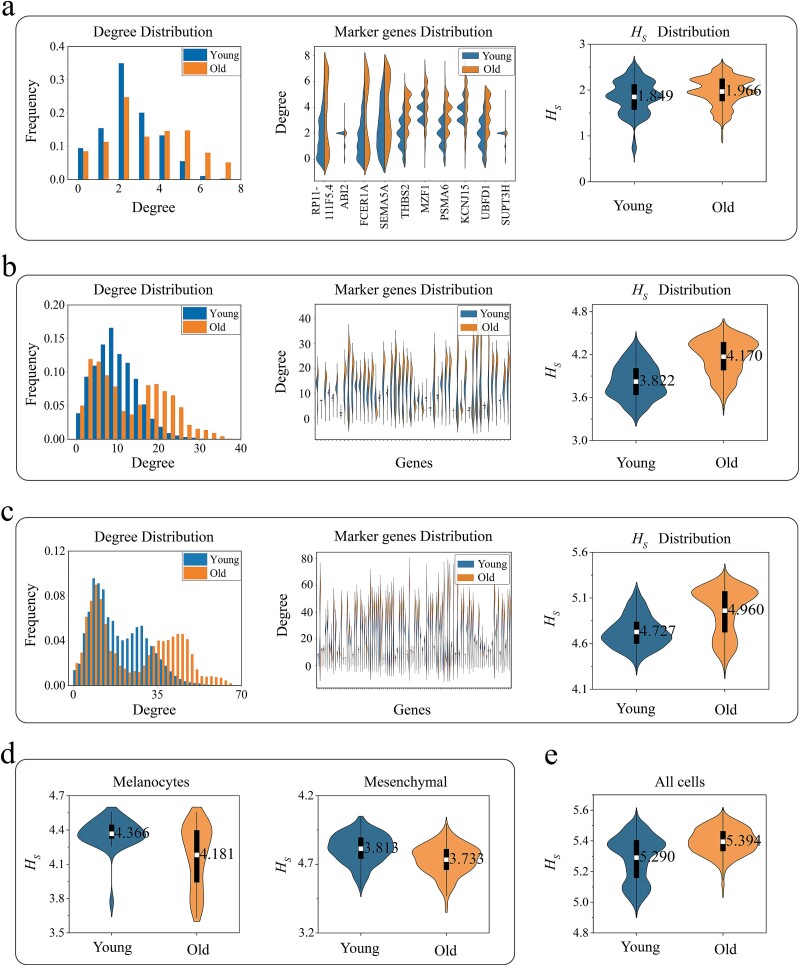
The distribution of degree and gene network structural entropy. (a) Top 10 marker genes for macrophages + DC cells. (b) Top 50 marker genes for macrophages + DC cells. (c) Top 100 marker genes for macrophages + DC cells. (d) The distribution of gene network structural entropy for the top 50 marker genes in melanocytes and mesenchymal cells. (e) The distribution of gene network structural entropy across all cell types.

Additionally, we also specifically calculated the degree and entropy distributions of the weighted correlation networks for 10, 50, and 100 marker genes in macrophages + DC cells. The results on the left side of Fig. 6 indicate that the degree distribution of aged macrophages + DC cells is broader and more diverse, suggesting the presence of more nodes with high connectivity in the network. Figure S6b further confirms this, showing that the mean and standard deviation of the degree in aging macrophages + DC cells significantly increase, revealing an increase in the variability and instability of gene interactions. The degree distributions of specific gene nodes in the middle sections of Fig. 6 provide more concrete evidence of this phenomenon. Genes such as RP11-111F5.4, FCER1A, and SEMA5A exhibit higher connectivity in aging macrophages + DC cells. Moreover, the gene network structural entropy of aging macrophages + DC cells is also relatively higher, indicating that their network structure is more complex and disordered. Studies have shown that with aging, macrophages in the immune system may be subject to more diverse and persistent extracellular signals, which may disrupt coordinated immune gene expression, resulting in age-related circadian and homeostatic immune dysfunction [79]. Aging macrophages have reduced calcium uptake, which triggers an inflammatory response [80]. This suggests that the GRN in aging cells may not respond to external stimuli in an orderly manner. These observations suggest that during aging, macrophages + DC cells may experience changes or losses in the function of key genes, and the precision of their regulatory mechanisms may be compromised, which may reflect a reduction in adaptability. Studies have shown that macrophages play a critical role in tissue regeneration, and aging can lead to a decline in tissue regenerative function, affecting the effective repair of tissue damage [57, 81].

Finally, we further explored the changes in GRN of other cell types during aging. We selected 50 marker genes as the basis and calculated the distribution of network structural entropy in different cell types. As shown in Fig. S7, we observed an increasing trend in network structural entropy in aged differentiated keratinocytes, pericytes, secretory-papillary cells, and secretory-reticular cells. This phenomenon suggests that as these cells age, their GRNs are tending toward a more disordered state, which may be accompanied by potential loss and disorder of genomic information and function [45, 82–84]. Meanwhile, in Fig. S7, we observed that the gene network structural entropy of Epithelial Stem Cells (EpSCs) and undifferentiated progenitors, T cells, and vascular Endothelial Cells (EC) did not exhibit significant changes. This suggests that these cells may maintain a certain degree of gene interaction patterns during aging, thereby preserving the stability of gene function [43]. For example, the maintenance of T-cell homeostasis is the key to the body’s resistance to aging and the maintenance of health [85–87].

Notably, we observed an interesting phenomenon in aged melanocytes and mesenchymal cells: The structural entropy of gene networks in these aged cells showed a decreasing trend compared to young cells, as shown in Fig. 6d. This decrease in network structural entropy indicates that the gene network patterns become more simplified and ordered. Figures S6c, S8, andS9 in the supplementary information provide strong evidence that the mean and standard deviation of the network degree in aging melanocytes significantly decrease, and the degree distribution becomes more concentrated and uniform, indicating a reduction in the complexity and uncertainty of the gene regulatory network. The orderliness of this gene network suggests that these cells are more inclined to perform specific functions during aging, rather than exhibiting the higher functional diversity and plasticity as young cells do. For example, aging melanocytes show reduced differentiation ability and plasticity, with a higher degree of differentiation, greater specificity, and more functional specialization [88]. Moreover, this process may also involve functional decline and loss, such as the decrease in the ability of melanocytes to maintain skin pigmentation balance and resist UV damage during aging [89]*.*

Furthermore, we analyzed the overall gene network structural entropy of all cells. As the fundamental units of life, cells need maintain a highly ordered state of function and structure [90]. However, as the organism ages, the molecular mechanisms and structures within cells may gradually deteriorate. This deterioration may be accompanied by metabolic disorders and functional failures, leading to an increase in the entropy (i.e. disorder) within the cells [91, 92]. In Fig. 6e, we present the changes in gene network structural entropy during aging across all types of cells, which are consistent with the above viewpoint. Our findings suggest that the changes in gene network structural entropy during aging are complex.

## Discussion and conclusions

Aging exhibits significant heterogeneity, with different individuals, tissues, cell types, and even processes within cells showing distinct aging patterns [93, 94]. Studies have revealed remodeling and functional changes in cellular networks during aging [95–97]. These studies indicate that aging not only affects the internal gene network structure of cells but also alters the regulatory relationships between genes, which may be maintained, enhanced, or weakened, in turn affecting specific or general biological functions of cells [98, 99]. Furthermore, the transcriptional profile of gene pathways changes with aging, and this change shows differences across cell populations and is regulated in a cell type–specific manner [93]. These findings highlight the diversity of different cell types in performing distinct functions and the complexity of gene regulation during aging. Traditional gene network methods often overlook the heterogeneity among different cell types, treating tissues or cell populations as homogeneous entities [100], which may restrict our understanding of the comprehensiveness of GRN. The advent of scRNA-seq allows for a deeper understanding of cellular network systems at the single-cell level [101, 102]. In this study, we used the method of single-cell gene importance ranking to construct weighted gene correlation networks related to aging to reveal the complex heterogeneity and dynamic changes within cell populations during aging.

Because relying solely on gene correlation network analysis may to some extent lose information about gene expression, we adopted an approach that simultaneously considers gene expression and gene networks to generate the GIM. GIM not only improves the clustering performance of cell types but also successfully reveals “dark” genes that are difficult to discover in traditional gene expression analysis, as shown in Fig. S5. These genes may not exhibit significant differences in expression levels but could play a pivotal role in the gene network, significantly influencing network regulation. Current research has targeted these “dark” genes to treat various diseases, including neurodegenerative diseases, cancer, autoimmune diseases, and aging [103]. Our research provides new insights in identifying and understanding these frequently neglected “dark” genes.

Although researchers have extensively studied cell fate through gene expression analysis, the cell fate is not only determined by the gene expression level but also controlled by the underlying GRN [104]. Currently, our understanding of age-related changes in cellular networks remains limited. To delve deeper into this issue, we constructed cell-type or state-specific weighted GRN within individual samples. The results in Fig. 4 and Fig. S10 demonstrate that during aging, not only do different types of cells exhibit heterogeneity, but even cells of the same type show heterogeneity between young and aged states. Compared to gene expression from single-cell data, the cell-specific networks we constructed provide a reliable characterization of the transition process of aging in different cell types.

The dynamism of GRN is a key mechanism by which cells respond to external stimuli and maintain the balance of cellular functions [3]. Therefore, a deep understanding of the changes in these networks is crucial for revealing the molecular mechanisms underlying the disease occurrence. In this study, we introduced the concept of network entropy to quantify the uncertainty or disorderliness of node connection patterns within the network [105]. Previous studies have utilized network entropy to quantify the differentiation potential of single cells and distinguish between normal and cancer stem cell phenotypes [106–108]. Studies have also shown that cells with lower degrees of differentiation exhibit higher “disorder” in their intracellular states [109]. In this study, we observed complex changes in the network structural entropy of cells during aging, which may be closely related to the different functions performed by these cells. As shown in Fig. 6, the network structural entropy of aging macrophages + DC cells is relatively high, which is consistent with the increased inflammatory levels and dysfunctional phenomena of macrophages during aging [30, 80]. Notably, the results in Fig. 6d show a decrease in the network structural entropy of aging melanocytes and mesenchymal cells, which contradicts our conventional understanding of entropy increase in biological systems. Typically, as organisms age, the intracellular molecules and structures may gradually degrade, and metabolic processes become more disordered, leading to an increase in entropy [90–92]. The changes in network structural entropy of all cells shown in Fig. 6e support this view. Here, the decrease in network structural entropy in aged cells may suggest a reduction in nonessential gene expression during aging, thereby simplifying the gene network. This simplification might prompt cells to adopt more energy-efficient regulatory mechanisms, reducing energy consumption in complex regulatory processes and maintaining core functions by optimizing gene expression patterns.

In summary, the quantification of gene network structural entropy provides a powerful tool for measuring the heterogeneity of gene networks. This indicator could help us identify complex network reconstructions that may occur during aging. Importantly, our study highlights the complexity of entropy changes during aging: Entropy can increase, remain unchanged, or decrease, and these changes are closely related to the functions of different cell types. By analyzing the changes in gene network structural entropy, we gain insights into the dynamic evolution of GRN from young to old age. Through this method, we can better understand the functional changes in cells during aging and provide new strategies for slowing the aging and treating age-related diseases.

**Key Points**

- The weighted gene correlation network integrating gene expression and gene network information was constructed by using single-cell RNA sequencing technology, providing a new analytical perspective for the study of cell aging.
- The Gene Importance Matrix was obtained based on the access probability of gene nodes by applying a random walk model. The Gene Importance Matrix enhanced cell type clustering performance and identified key genes involved in the aging process.
- The complexity of gene networks during aging was quantitatively analyzed by introducing network structural entropy. It was found that the overall network structural entropy of aging cells exhibited an increasing trend, while different functional cell subtypes showed diverse changes.

## Supplementary Material

SUPPLEMENTARY_INFORMATION_bbae698

## Data Availability

The source code and the data are available at https://github.com/LiuZhilong-Biophysics/Gene-Network-Entropy. The raw data [45] can be accessed from Gene Expression Omnibus (GEO) through the accession number GSE130973.
