# Supplementary material for: The network structural entropy for single-cell RNA sequencing data during skin aging
Source: Brief Bioinform. 2025 Jan 5;26(1):bbae698. doi: 10.1093/bib/bbae698 (PMC11700662; doi:10.1093/bib/bbae698)
Supplement: SUPPLEMENTARY_INFORMATION_bbae698 [file supplementary_information_bbae698.zip › SUPPLEMENTARY INFORMATION.docx]

**SUPPLEMENTARY INFORMATION**

The network structural entropy for single-cell RNA sequencing data during skin aging

Zhilong Liu, Hai Lin, Xiang Li, Hao Xue,Yuer Lu, Fei Xu, Jianwei Shuai

1. **Additional Methodological Details**

Specifically, if the value of 𝜌_𝑖𝑗_^𝑘^​ exceeds the significance level 𝛼_𝑖𝑗_ ​, it indicates significant non-independence between the expression levels of genes *i* and *j* in cell *k*. In this study, the significance level 𝛼_𝑖𝑗_​ is set to 0.01. Therefore, in the gene correlation matrix *A* of cell *k*, the correlation between gene *i* and gene *j*, *A_ij_^k^*, is defined as:

*__* (S1)

Within the analytical framework of single-cell gene correlation networks, we can consider the gene correlation network as a vast network system, where the function of each gene node is analogous to that of a web page on the Internet. The importance of each gene node can thus be quantified using the PageRank algorithm. This algorithm follows a random walk model, simulating a process of random wandering within the network, where the transition from one gene node to the next is based on the internal associations of the network and is conducted randomly without being influenced by the previous path. This random walk characteristic aligns with the properties of a Markov chain, which is a stochastic process where the transition probability to future states depends only on the current state and is independent of past states. In the PageRank algorithm, we assume that the transition probabilities between genes are equal, meaning that the probability of each gene node being visited during the random walk process is uniform. The PageRank score is calculated by the following formula:

$PR(T+1)_{i}^{k}=(1-d)+d\sum_{j\in L_{i}^{k}} \frac{PR(T)_{j}^{k}}{N_{j}^{k}}$ (S2)

Where T represents the number of iterations, *PR*(T+1)*_i_^k^* ​ denotes the PageRank score of gene *i* in cell *k* at iteration T+1. The damping factor *d* is fixed at 0.85(46). *L_i_^k^* ​ represents the set of neighboring genes of gene *i*, *N_j_^k^* denotes the number of genes associated with gene *j* in cell *k*, and *PR*(T)*_j_^k^*​ denotes the PageRank score of gene *j* in cell *k* at iteration T. The initial value for the *PR* iteration is set to 1/𝑛_𝐺_​​, where 𝑛_𝐺_ is the total number of genes. The iteration will continue until the *PR* reaches a stable distribution. The basic assumption of PageRank is that the initial probability of a node (in this study, a gene) visiting other linked nodes is equal. As the iterations proceed, the network's connectivity will lead to a redistribution of these probabilities. Eventually, the visit probability for each node stabilizes at a fixed value, which reflects the node's importance in the network. In this model, popular nodes, those connected to many other nodes, will have a higher probability of being visited. Therefore, a gene with many correlation edges in the network or connections to multiple key gene nodes is often biologically more significant. The advantage of this method lies in its unbiased nature, as it does not rely on predefined gene function classifications or specific biological assumptions, instead inferring gene importance directly from the data.

In the normalized network, the edges between gene pairs are represented by correlation probabilities, as shown in Fig.1e. The correlation probability between gene pairs is calculated by the following formula:

$P_{ij}^{M}=\frac{1}{N^{M}}\sum_{k=1}^{N^{M}} A_{ij}^{k}$, (S3)

where $P_{ij}^{M}$ represents the correlation probability between gene i and gene j in a specific cell type M, $N^{M}$ denotes the total number of cells in the specific cell type, and $A_{ij}^{k}$ is obtained from Equation (S1).

1. **Additional Details for Results**

Fig.S1. Visualization of single-cell GEM for 11 other cell types. The cell types include: Diff. Keratinocytes, Mesenchymal, EpSC and undiff. Progenitors, Erythrocytes, Lymphatic EC, Melanocytes, Pericytes, Secretory-papilliary, Secretory-reticular, T cells and Vascular EC.

Based on the preprocessed GEM, we performed clustering analysis on young and aging cells of different types of cell populations to reveal their differences. The results of Fig.S1 show that the clusters of aging and young cells appear relatively dispersed, which indicate that the distinction between aging and young cells is not very pronounced.

Fig.S2. Visualization of single-cell GIM for 11 other cell types. The cell types include: Diff. Keratinocytes, Mesenchymal, EpSC and undiff. Progenitors, Erythrocytes, Lymphatic EC, Melanocytes, Pericytes, Secretory-papilliary, Secretory-reticular, T cell and Vascular EC.

Based on GIM, we performed clustering analysis on young and aging cells of different types of cell populations. Visualization in Fig. S2 demonstrates that clusters of aging or young cells appear more tightly grouped, which indicate that GIM enhances the distinction between young and aging cells.

Fig.S3. Performance comparison between GIM and GEM. (a), (b), (c) targeted Pro-inflammatory cells, Macrophages+DC cells, and all cell types. The performance of GIM is compared with GEM by ARI. (d) (e) (f) targeted Pro-inflammatory cells, Macrophages+DC cells, and all cell types. The performance of GIM is compared with GEM by NMI.

In the combination spaces of PC1 vs. PC2, PC1 vs. PC3, PC1 vs. PC4, PC2 vs. PC3, PC2 vs. PC4, and PC3 vs. PC4, GIM performs significantly better than GEM in distinguishing young and aging cells of Pro-inflammatory cells and Macrophages + DC cells. To further validate the robustness of our method, we calculated ARI and NMI for all 13 cell types involved in the study, with the results shown in parts (c) and (f) of Figure R2. The results indicate that, for both median and mean values of ARI and NMI, GIM consistently outperforms GEM. Additionally, the results revealed that GIM's performance, both in terms of ARI and NMI, is particularly outstanding in the PC2 vs. PC4 combination space, surpassing other principal component combinations.

Fig.S4. ARI, FMI and NMI performance comparison between GIM, NDM and GEM. (a) Pro-inflammatory cells. (b) Macrophages+DC cells.

We compared the Gene Correlation Network Degree Matrix (NDM) method and applied PCA dimensionality reduction to the GEM, NDM, and GIM datasets, followed by the calculation of the Pro-inflammatory cells and Macrophages + DC cells datasets. Using the Adjusted Rand Index (ARI), Fowlkes-Mallows Index (FMI), and Normalized Mutual Information (NMI) as evaluation metrics, we compared the clustering results with the original cell type labels, as shown in Figure S4. The results demonstrate that GIM exhibits superior performance.

Fig. S5. Comparison of marker genes of expression and importance. (a) Performance of gene RP11-315120.1 and gene CHM in PCA for Pro-inflammatory cells. (b) Performance of gene MT1F and gene KIAA0513 in PCA for Macrophages+DC cells.

Gene RP11-315120.1 and gene CHM show insignificant expression in both young and aging Pro-inflammatory cells. However, gene RP11-315120.1 may play an important role in young cells, while gene CHM might be important in aging cells. Similarly, gene MT1F and gene KIAA0513 exhibit insignificant expression in both young and aging Macrophages+DC cells. However, gene KIAA0513 may play a crucial role in young cells, whereas gene MT1F might be significant in aging cells.

Fig.S6. Analysis of mean and standard deviation of degree for Top 50 Marker genes in young and aging cells. (a) Pro-inflammatory cells. (b) Macrophages+DC cells. (c) Melanocytes cells.

For aging pro-inflammatory cells, there was no significant change in the mean and standard deviation of gene node degrees compared to young cells. In contrast, aging Macrophages and DC cells (Macrophages+DC) exhibited a notable increase in both the mean and standard deviation of gene node degrees. Conversely, aging Melanocytes showed a significant decrease in both the mean and standard deviation of gene node degrees.

Fig.S7. Distribution of gene network structural entropy for 9 other cell types. Cell types include: Diff. Keratinocytes, Pericytes, Secretory-papilliary, Secretory-reticular, EpSC and undiff. Progenitors, Vascular EC, T cells, Lymphatic EC and Erythrocytes.

Young and aging states of cell types exhibit distinct distributions of gene network structural entropy. The patterns of gene network structural entropy in young and aging cells display different states, which reveal not only intrinsic specificities of cell types but also reflect cellular heterogeneity during aging. Specifically, Diff. Keratinocytes, Pericytes, Secretory-papillary, and Secretory-reticular cells show an increase in gene network structural entropy during aging. In contrast, EpSC and undiff. Progenitors, Vascular Endothelial and T cells maintain relatively stable gene network structural entropy during aging. Meanwhile, Lymphatic EC and Erythrocytes demonstrate a decreasing trend in gene network structural entropy with aging.

Fig.S8. Gene network structural entropy of Melanocytes cells. (a) Top 10 marker genes. (b) Top 50 marker genes. (c) Top 100 marker genes. The left panel shows the frequency of the degree of gene nodes in the network, the middle panel displays the distribution of the degree of gene nodes, and the right panel shows the distribution of gene network structural entropy between young and aging cells. The white box and number in the violin plots represent the mean value.

Fig.S9. Gene network structural entropy of Mesenchymal cells. (a) Top 10 marker genes. (b) Top 50 marker genes. (c) Top 100 marker genes. The left panel shows the frequency of the degree of gene nodes in the network, the middle panel displays the distribution of the degree of gene nodes, and the right panel shows the distribution of gene network structural entropy between young and aging cells. The white box and number in the violin plots represent the mean value.

Fig.S10. Gene networks constructed from Top 10 Marker genes for 11 other cell types. Cell types include: Diff. Keratinocytes, Mesenchymal, Vascular EC, EpSC and undiff. Progenitors, Erythrocytes, T cells, Lymphatic EC, Melanocytes, Secretory-reticular, Pericytes and Secretory-papilliary.

There exists significant network heterogeneity among different cell types, which reflect their unique biological characteristics and regulatory mechanisms. Even within the same cell type, differences in network heterogeneity between young and aging states are observed, which reflect cellular responses and adaptive changes to aging.
