# Supplementary figures and images for: The network structural entropy for single-cell RNA sequencing data during skin aging
Source: Brief Bioinform. 2025 Jan 5;26(1):bbae698. doi: 10.1093/bib/bbae698 (PMC11700662; doi:10.1093/bib/bbae698)

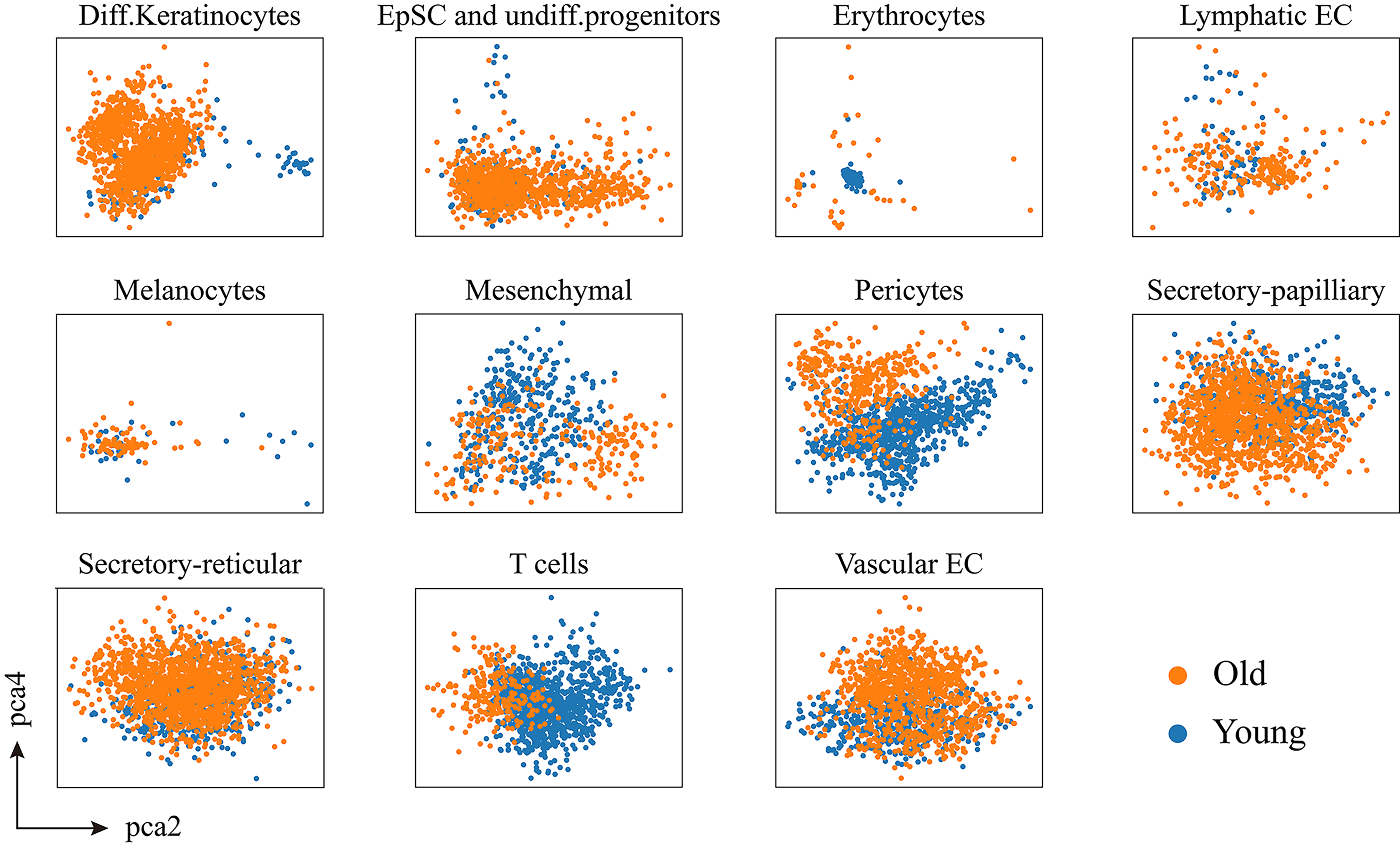

Supplement: SUPPLEMENTARY_INFORMATION_bbae698 [file supplementary_information_bbae698.zip › Fig S1.tif]

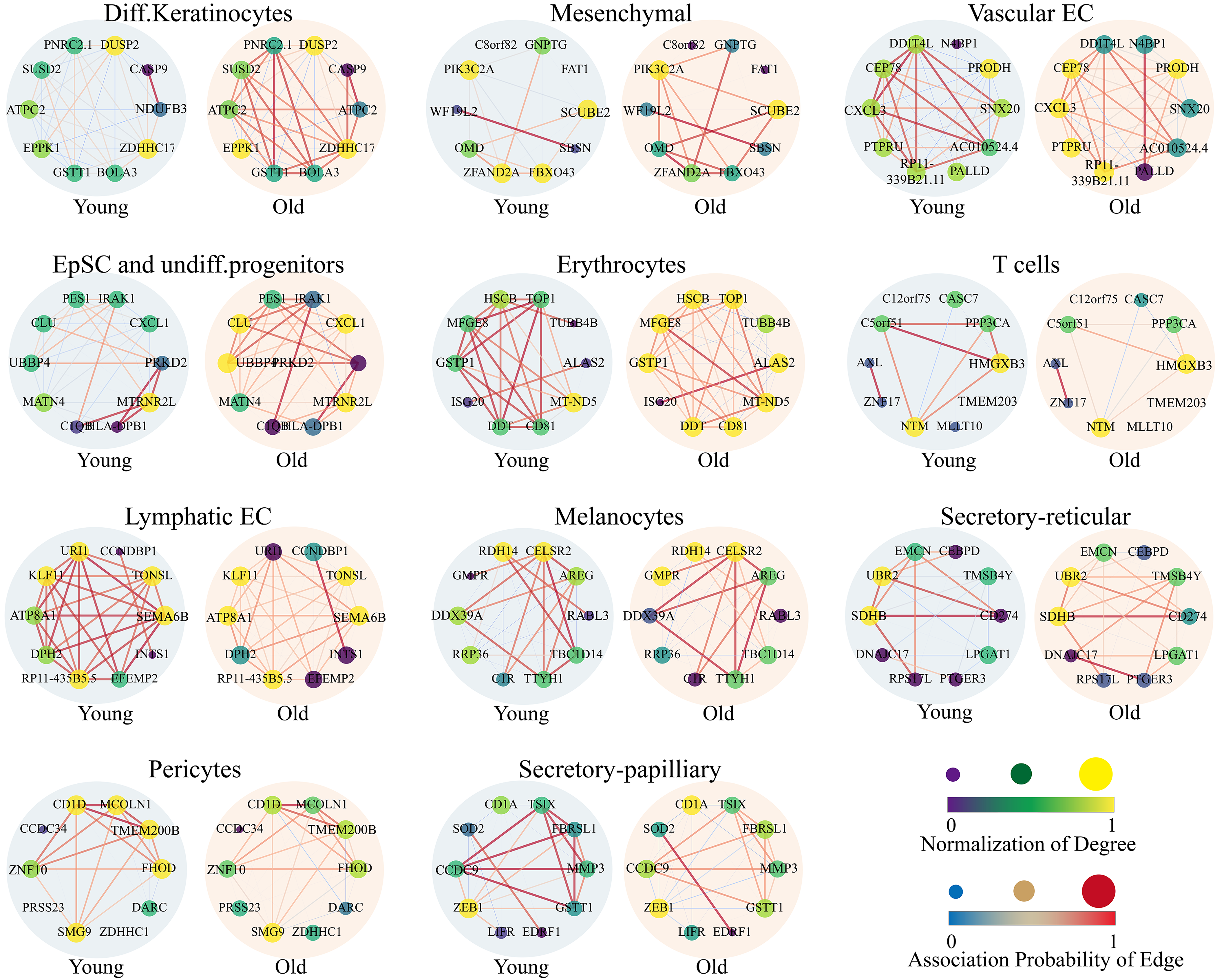

Supplement: SUPPLEMENTARY_INFORMATION_bbae698 [file supplementary_information_bbae698.zip › Fig S10.tif]

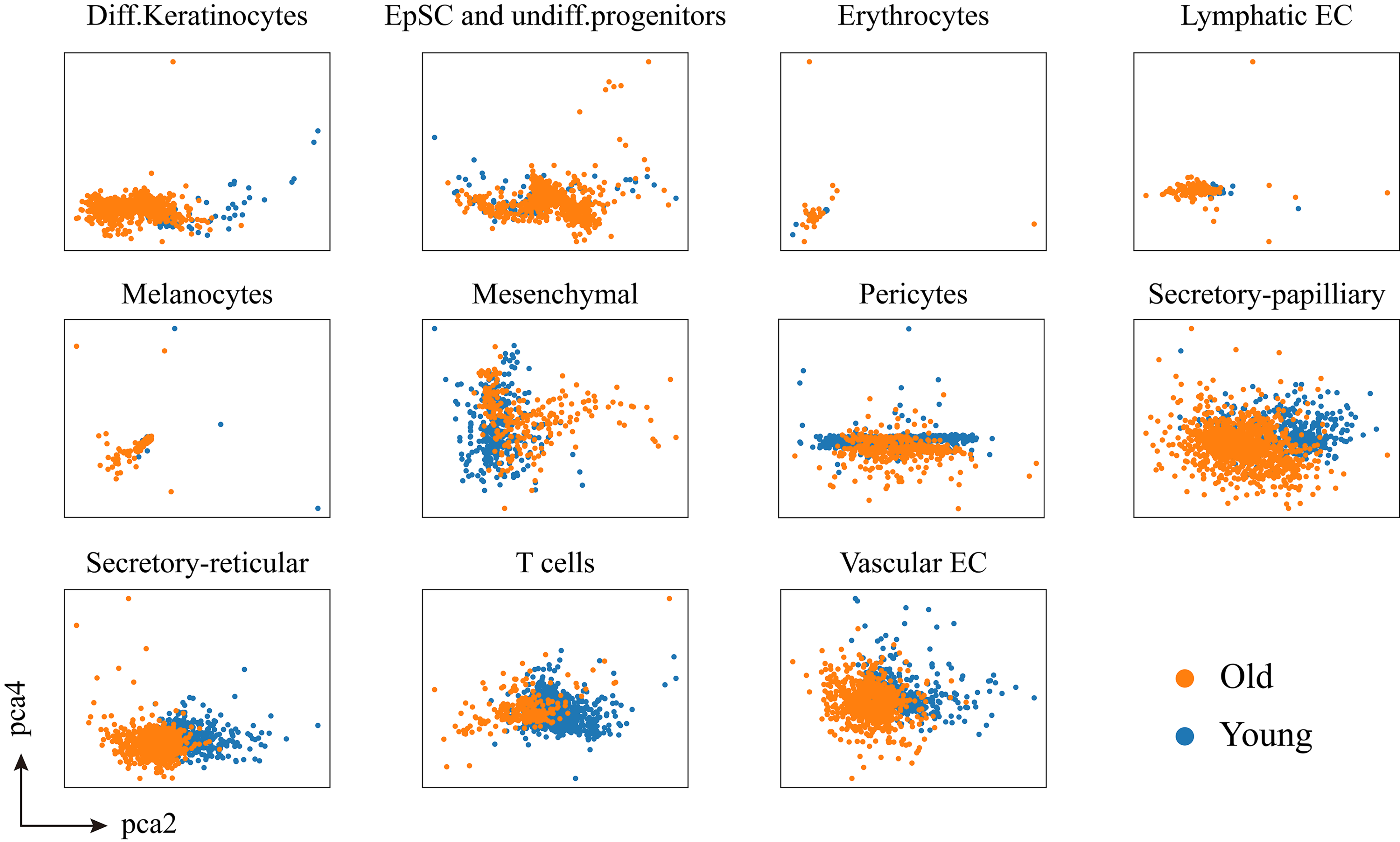

Supplement: SUPPLEMENTARY_INFORMATION_bbae698 [file supplementary_information_bbae698.zip › Fig S2.tif]

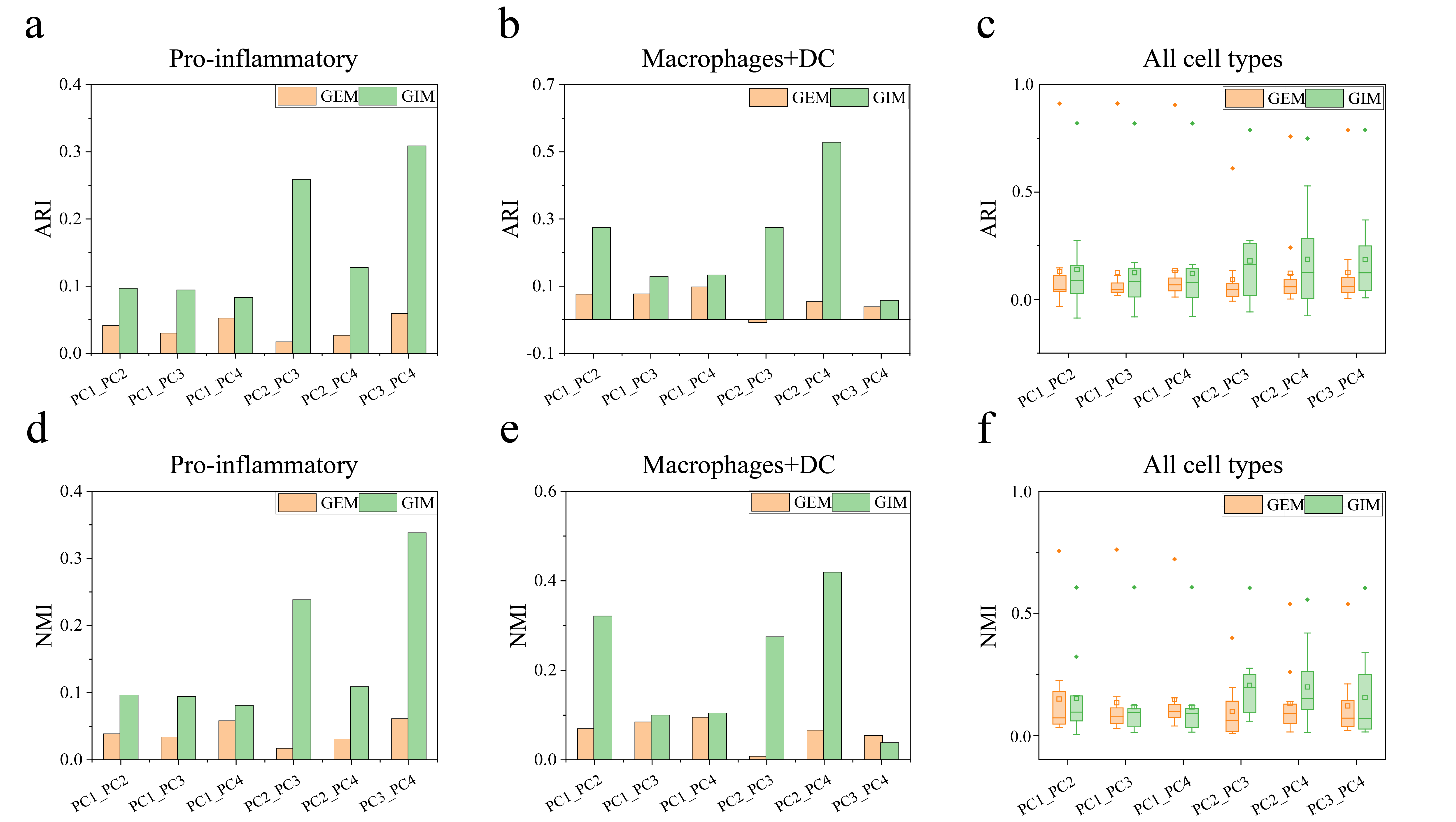

Supplement: SUPPLEMENTARY_INFORMATION_bbae698 [file supplementary_information_bbae698.zip › Fig S3.tif]

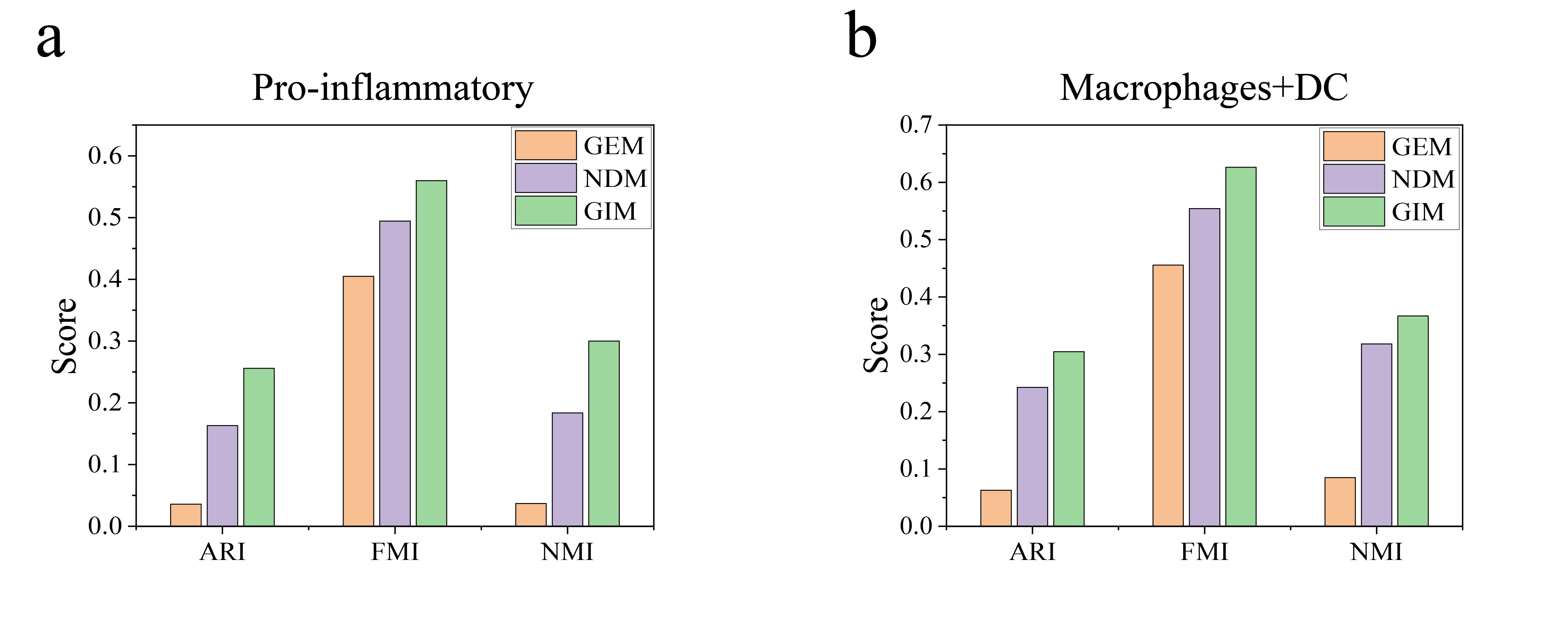

Supplement: SUPPLEMENTARY_INFORMATION_bbae698 [file supplementary_information_bbae698.zip › Fig S4.tif]

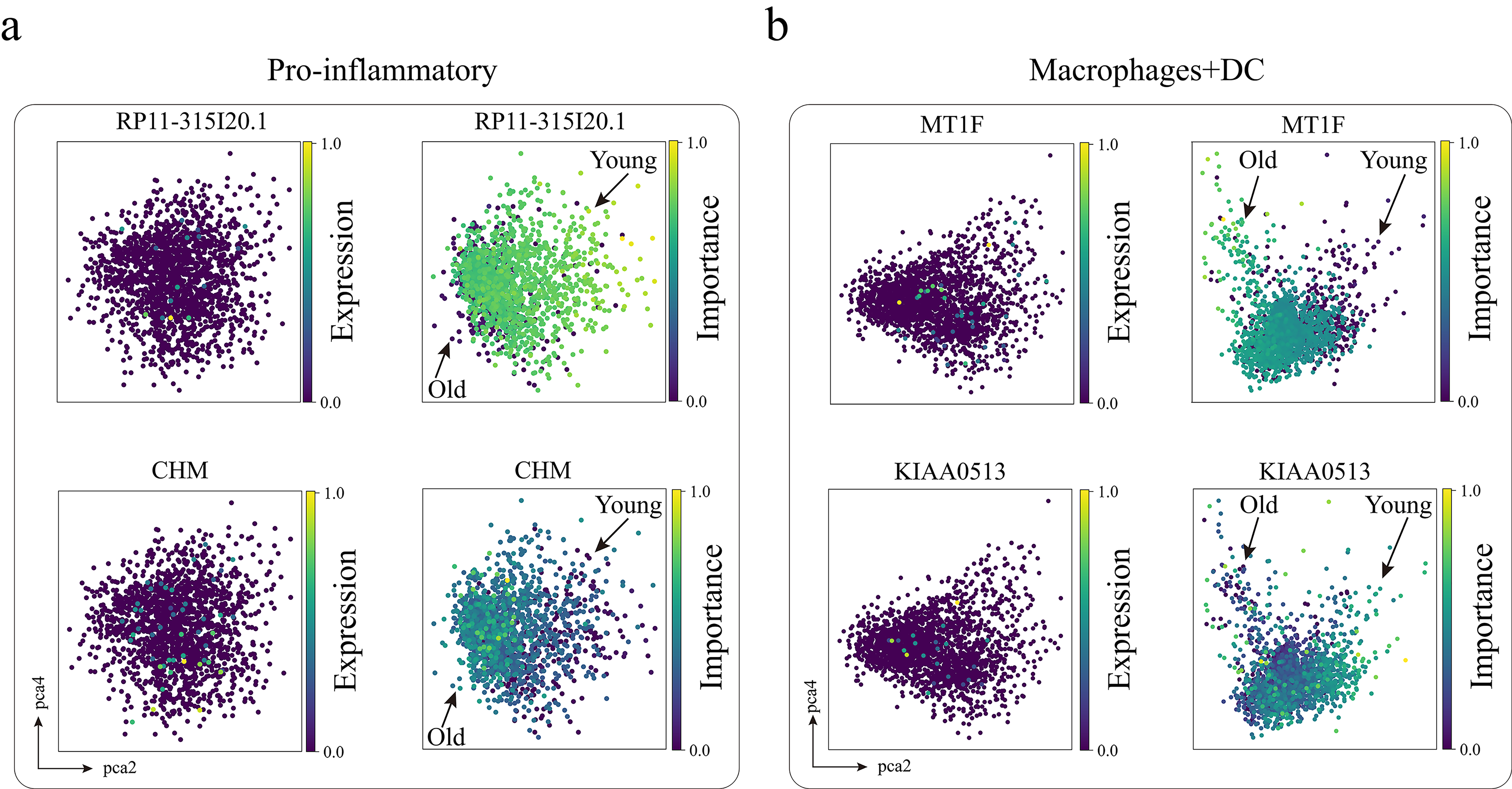

Supplement: SUPPLEMENTARY_INFORMATION_bbae698 [file supplementary_information_bbae698.zip › Fig S5.tif]

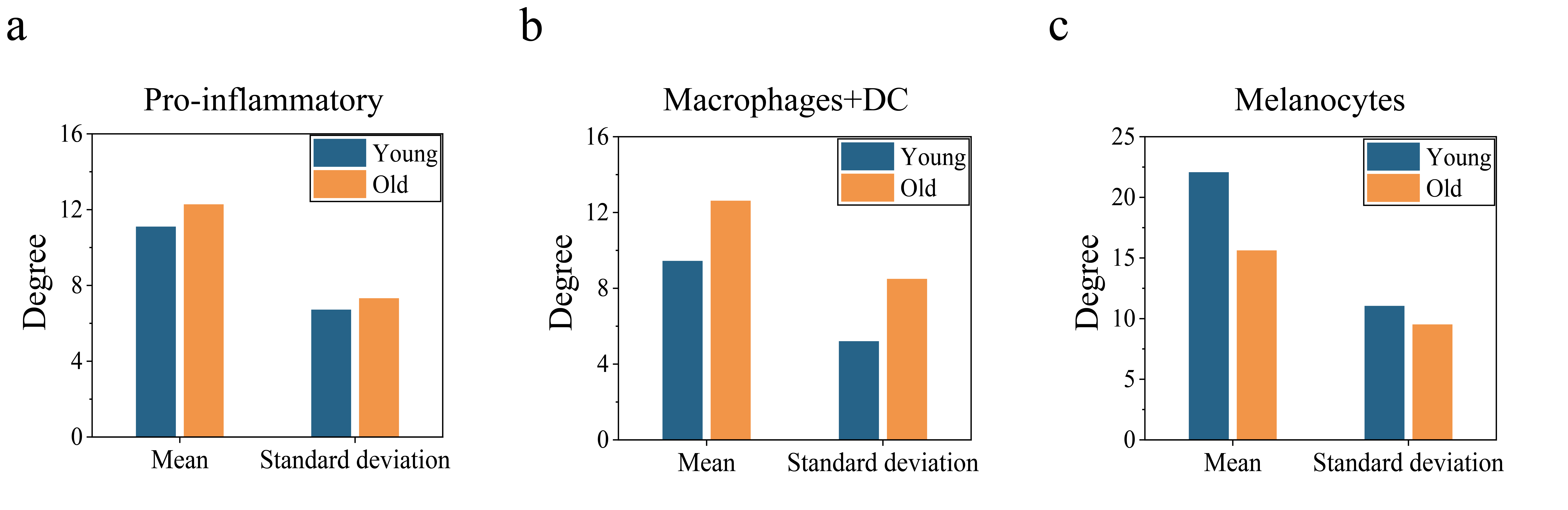

Supplement: SUPPLEMENTARY_INFORMATION_bbae698 [file supplementary_information_bbae698.zip › Fig S6.tif]

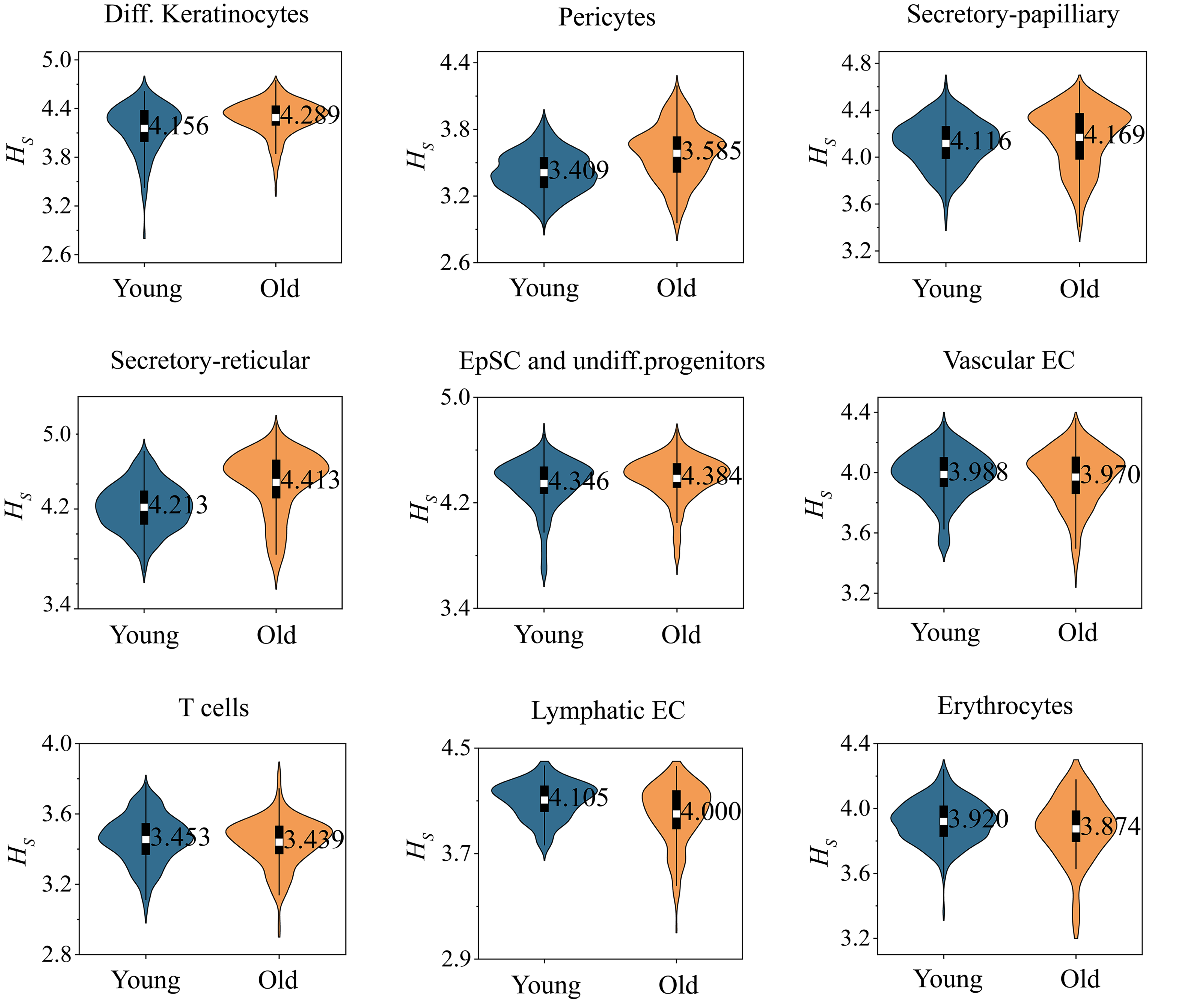

Supplement: SUPPLEMENTARY_INFORMATION_bbae698 [file supplementary_information_bbae698.zip › Fig S7.tif]

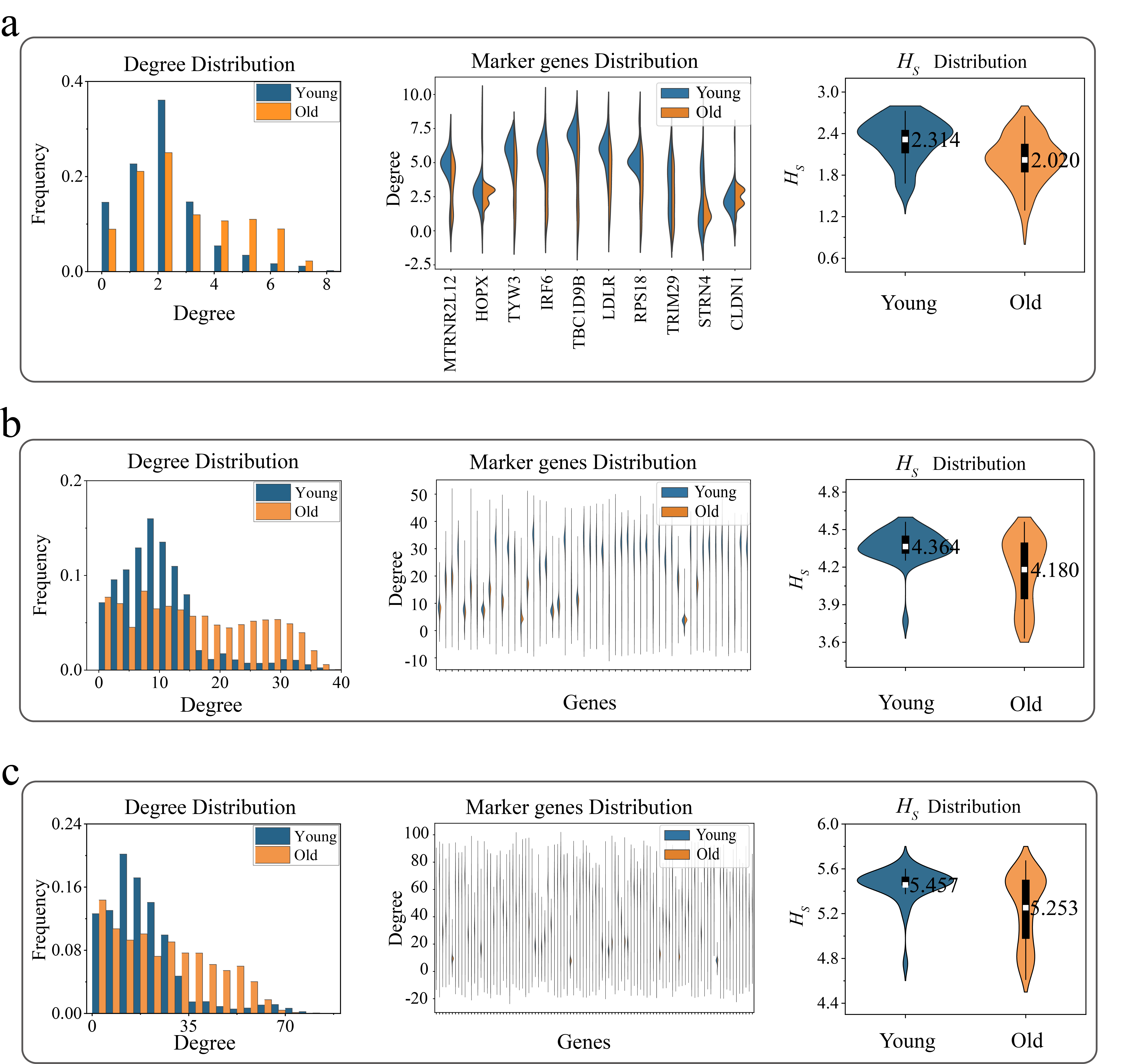

Supplement: SUPPLEMENTARY_INFORMATION_bbae698 [file supplementary_information_bbae698.zip › Fig S8.tif]

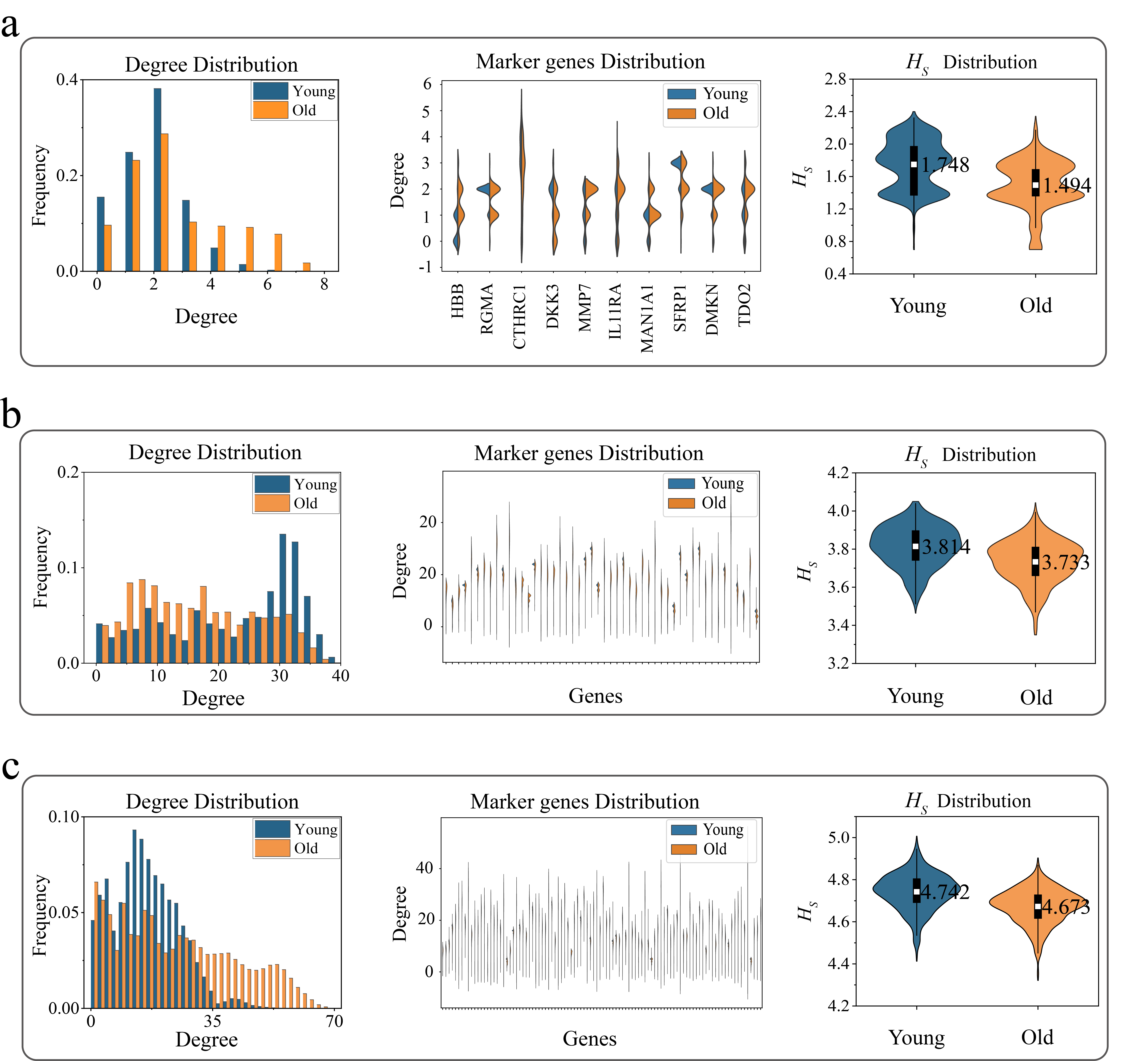

Supplement: SUPPLEMENTARY_INFORMATION_bbae698 [file supplementary_information_bbae698.zip › Fig S9.tif]
